# Health worker protests and the COVID-19 pandemic: an interrupted time-series analysis

**DOI:** 10.2471/BLT.23.290330

**Published:** 2024-07-04

**Authors:** Kartik Sharma, Sorcha Brophy, Michael Law, Veena Sriram

**Affiliations:** a Centre for Health Services and Policy Research, School of Population and Public Health, University of British Columbia, 201-2206 East Mall, Vancouver, BC V6T 1Z3, Canada.; b Mailman School of Public Health, Columbia University, New York, United States of America.; c School of Public Policy and Global Affairs, University of British Columbia, Vancouver, Canada.

## Abstract

**Objective:**

To assess the impact of the coronavirus disease 2019 (COVID-19) pandemic on protests by health workers.

**Methods:**

We conducted an interrupted time series analysis of data from 159 countries for 2 years before and after the World Health Organization classified COVID-19 as a pandemic in March 2020, thus between 2018 and 2022. We produced models examining two main outcomes: (i) the total weekly number of health worker protests globally; and (ii) the number of countries with one or more health worker protests in a given week.

**Findings:**

In total, there were 18 322 health worker protests in 133 countries between 2018 and 2022. The number of weekly health worker protests globally increased by 47% (30.1/63.5), an increase of 30.1 protests per week (95% confidence interval, CI: 11.7–48.6) at the onset of the COVID-19 pandemic. Furthermore, the number of countries experiencing such protests in a given week increased by 24% (5.7/24.1) following the declaration of the pandemic (an increase of 5.7 countries; 95% CI: 3.5–7.8).

**Conclusion:**

The pandemic increased the overall level of health worker protests globally as well as the number of countries experiencing such protests. These protests highlight discontent in the health workforce. Given the ongoing global health workforce crisis, understanding and addressing the drivers of health worker discontent is important for global health policy and security.

## Résumé

**Objectif:**

Évaluer l'impact de la pandémie de maladie à coronavirus 2019 (COVID-19) sur les manifestations du personnel de santé.

**Méthodes:**

Nous avons effectué une analyse de série chronologique interrompue de données issues de 159 pays deux ans avant et deux ans après la classification, par l'Organisation mondiale de la Santé, de la COVID-19 dans la catégorie des pandémies en mars 2020, donc entre 2018 et 2022. Nous avons ensuite élaboré des modèles pour deux éléments principaux: (i) le nombre total de manifestations organisées chaque semaine par le personnel de santé; ainsi que (ii) le nombre de pays où le personnel de santé a manifesté au moins une fois au cours d'une semaine donnée.

**Résultats:**

Le personnel de santé a participé à un total de 18 322 manifestations dans 133 pays entre 2018 et 2022. Le nombre de manifestations hebdomadaires a augmenté de 47% (30,1/63,5) à l'échelle planétaire, l'équivalent de 30,1 manifestations supplémentaires par semaine (intervalle de confiance de 95%, IC: 11,7–48,6) au moment de l'apparition de la pandémie de COVID-19. En outre, le nombre de pays touchés par ces manifestations au cours d'une semaine donnée a augmenté de 24% (5,7/24,1) lorsque l'état de pandémie a été déclaré (ce qui représente une hausse de 5,7 pays; IC de 95%: 3,5–7,8).

**Conclusion:**

La pandémie a entraîné une recrudescence des manifestations organisées par le personnel de santé dans le monde, ainsi que du nombre de pays confrontés à de telles manifestations, ce qui illustre le mécontentement qui règne au sein du secteur. Étant donné la crise que connaît actuellement le personnel de santé partout dans le monde, s'intéresser et s'attaquer aux facteurs de mécontentement des soignants est essentiel pour la sécurité et la politique en matière de santé mondiale.

## Resumen

**Objetivo:**

Evaluar el impacto de la pandemia de la enfermedad por coronavirus de 2019 (COVID-19) en las protestas del personal sanitario.

**Métodos:**

Se realizó un análisis de series temporales interrumpidas de datos de 159 países durante dos años antes y después de que la Organización Mundial de la Salud clasificara la COVID-19 como pandemia en marzo de 2020, es decir, entre 2018 y 2022. Se elaboraron modelos para examinar dos resultados principales: (i) el número total semanal de protestas del personal sanitario en todo el mundo; y (ii) el número de países con una o más protestas del personal sanitario en una semana determinada.

**Resultados:**

En total, hubo 18 322 protestas de personal sanitario en 133 países entre 2018 y 2022. El número de protestas semanales del personal sanitario en todo el mundo aumentó un 47% (30,1/63,5), lo que supone un aumento de 30,1 protestas por semana (intervalo de confianza del 95%, IC: 11,7-48,6) al inicio de la pandemia de la COVID-19. Además, el número de países que experimentaron este tipo de protestas en una semana determinada aumentó un 24% (5,7/24,1) tras la declaración de la pandemia (un aumento de 5,7 países; IC del 95%: 3,5-7,8).

**Conclusión:**

La pandemia aumentó el nivel general de protestas del personal sanitario en todo el mundo, así como el número de países que las experimentaron. Estas protestas ponen de manifiesto el descontento del personal sanitario. Dada la actual crisis mundial del personal sanitario, comprender y abordar los factores que impulsan el descontento del personal sanitario es importante para la política y la seguridad sanitarias a nivel mundial.

## ملخص

**الغرض:**

تقييم تأثير جائحة مرض فيروس كورونا 2019 (كوفيد 19) على احتجاجات العاملين في المجال الصحي.

**الطريقة:**

قمنا بإجراء تحليل سلاسل زمنية متقطعة للبيانات من 159 دولة على مدار عامين، قبل وبعد أن قامت منظمة الصحة العالمية بتصنيف كوفيد 19 على أنه جائحة في مارس/آذار 2020، وبالتالي خلال الفترة من 2018 إلى 2022. وأنتجنا نماذجًا تفحص نتيجتين رئيسيتين: (1) العدد الإجمالي الأسبوعي لاحتجاجات العاملين في المجال الصحي على مستوى العالم؛ و(2) عدد الدول التي يحدث بها احتجاج واحد أو أكثر من العاملين في المجال الصحي في أسبوع محدد.

**النتائج:**

بشكل إجمالي، كان هناك 18322 احتجاجًا للعاملين في المجال الصحي في 133 دولة خلال الفترة من 2018 إلى 2022. ارتفع عدد الاحتجاجات الأسبوعية للعاملين في المجال الصحي على مستوى العالم بنسبة %47 (30.1/63.5)، بزيادة قدرها 30.1 احتجاجًا في الأسبوع (بفاصل ثقة مقداره %95: 11.7 إلى 48.6) مع اندلاع جائحة كوفيد 19. بالإضافة إلى ذلك، زاد عدد الدول التي تشهد مثل هذه الاحتجاجات في أسبوع محدد بنسبة %24 (5.7/24.1) بعد إعلان الجائحة (بزيادة قدرها 5.7 دولة؛ بفاصل ثقة مقداره %95: 3.5 إلى 7.8).

**الاستنتاج:**

أدت الجائحة إلى زيادة المستوى العام لاحتجاجات العاملين في المجال الصحي على مستوى العالم، وكذلك عدد الدول التي تمر بمثل هذه الاحتجاجات. تركز هذه الاحتجاجات على غضب القوى العاملة الصحية. في ظل الأزمة القائمة للقوى العاملة الصحية العالمية، فإن فهم دوافع غضب العاملين في المجال الصحي، والتعامل معها، لهو أمر مهم لسياسة الصحة العالمية والأمن.

## 摘要

**目的:**

旨在评估 2019 冠状病毒病 (COVID-19) 大流行疫情对卫生工作者抗议活动的影响**。**

**方法:**

在世界卫生组织于 2020 年 3 月将 COVID-19 列为一种大流行病之前和之后的 2 年内，即 2018 年至 2022 年期间，我们利用从 159 个国家收集的数据进行了中断时间序列分析。我们构建了模型来研究两种主要结果：（i）每周全球卫生工作者开展抗议活动的总数；以及（ii）在给定的一周时间内发生了一起或多起卫生工作者抗议活动的国家数量。

**结果:**

在 2018 年至 2022 年期间，133 个国家共发生了 18,322 起卫生工作者抗议活动。COVID-19 大流行疫情开始蔓延时，每周全球卫生工作者抗议活动的数量增加了 47% (30.1/63.5)，即每周增加 30.1 起抗议活动（95% 置信区间，CI：11.7–48.6）。此外，在宣布大流行疫情开始蔓延后，在给定的一周时间内发生此类抗议活动的国家数量增加了 24% (5.7/24.1)（增加了 5.7 个国家；95% CI：3.5–7.8）。

**结论:**

大流行疫情导致全球卫生工作者抗议活动的总数量增多，且发生此类抗议活动的国家数量也随之增多。这些抗议活动凸显了卫生工作者的不满情绪。由于全球卫生工作者危机持续存在，了解和解决导致卫生工作者不满的根本问题对实施全球卫生政策和确保全球卫生安全至关重要。

## Резюме

**Цель:**

Оценить влияние пандемии коронавирусной инфекции 2019 г. (COVID-19) на протесты медицинских работников.

**Методы:**

Был проведен анализ прерывистых временных рядов данных из 159 стран за 2 года до и после того, как Всемирная организация здравоохранения классифицировала COVID-19 как пандемию в марте 2020 года, то есть между 2018 и 2022 годами. Авторами были разработаны модели, изучающие два основных показателя: (i) общее еженедельное количество протестов медицинских работников в мире; (ii) количество стран, в которых в течение недели проводился один или несколько протестов медицинских работников.

**Результаты:**

В общей сложности в период с 2018 по 2022 год в 133 странах было проведено 18 322 акции протеста работников здравоохранения. Количество еженедельных протестов медицинских работников в мире увеличилось на 47% (30,1/63,5), то есть на 30,1 протеста в неделю (95%-й ДИ: 11,7–48,6) в начале пандемии COVID-19. Кроме того, после объявления пандемии число стран, в которых в течение недели проходили такие протесты, увеличилось на 24% (5,7/24,1) (увеличение на 5,7 страны; 95%-й ДИ: 3,5–7,8).

**Вывод:**

В результате пандемии увеличился общий уровень протестов медицинских работников во всем мире, а также число стран, в которых эти протесты имели место. Эти протесты свидетельствуют о недовольстве медицинских работников. С учетом продолжающегося глобального кадрового кризиса в сфере здравоохранения понимание и устранение причин недовольства медицинских работников имеет важное значение для глобальной политики в области здравоохранения и безопасности.

## Introduction

Protests, that is organized public demonstrations of grievances, by health workers have gained increasing attention in recent years. Health workforce crises are occurring around the world in both high- and lower-income countries because of staffing shortages, poor or dangerous working conditions, and conflicts over compensation and benefits.^1^^,^^2^ However, even though the protest activities of health workers have generated considerable interest, little is known about such protests on a global scale. Additionally, although health worker protests are presumed to have increased as a result of the coronavirus disease 2019 (COVID-19) pandemic,^3^ such claims lack empirical evidence.

Research on health worker protest activity has generally focused on individual protest events, and has largely been concerned with the effect of strikes on patient mortality, morbidity and other outcomes.^4^^–^^7^ These studies find mixed results about the impact of strikes (one of several forms of protest action) on patient outcomes. A 2022 meta-analysis found no significant effect of strikes on in-hospital or population mortality, although substantial heterogeneity existed across the studies.^7^ However, a focus only on the immediate impact on service delivery fails to address the structural problems being highlighted by health workers through protest activity.^8^ Public expressions of health worker grievances provide important data about underinvestment in health systems as well as insight into the ongoing workforce crisis. A few case studies have examined the relationship between health worker protests and structural failings of health systems.^9^^–^^13^ Complementing these case studies with insights on changes in health worker protest activity would be valuable as such information might highlight pandemic-related challenges within health systems. Viewed globally, data on the scale and trajectory of health worker protests could be used to draw conclusions about health-system preparedness for disasters, as well as the availability of settings where health workers can have a voice in policy processes.

To date, few studies have been published on health worker protests on a global scale, and even fewer have been published on the effect of COVID-19 on protest trends. Most of the multicountry analyses published were conducted before COVID-19 and used limited data sets.^14^^,^^15^ One study described health worker protests in 90 countries during the pandemic and suggested that protest activity spiked at its onset.^16^ However, this study lacked data from before the pandemic and was therefore unable to make any claims about the impact of the pandemic compared with previous trends. A 2022 study compared the number of health worker protests in 85 countries in the year before and year after the onset of the COVID-19 pandemic. The study used a simple pre–post analysis and identified a 62% (1497/2416) increase in protest activity.^17^ While the incorporation of data from before the pandemic is valuable, the use of only two point estimates to make this assessment means that it may be biased due to the presence of pre-existing trends in the data.

Overall, high-quality evidence is lacking on the effect of COVID-19 on trends in health worker protests. This lack of evidence means that claims about the effect of COVID-19 on protest activity are unsubstantiated and we are missing valuable information about the state of the global health workforce. In this study, we present empirical evidence to support claims about the effect of the pandemic on protests by health workers and provide a robust and global measurement of how COVID-19 affected trends in health worker protests.

## Methods

### Data sources and outcomes

We used data from the Armed Conflict Location and Event Data Project.^18^ This data set provides event-level details on political violence, demonstrations and non-violent mobilizations. Protests were the event of interest for our study and are defined by the Armed Conflict Location and Event Data Project as “in-person public demonstration[s] of three or more participants in which the participants do not engage in violence, though violence may be used against them. Events include individuals and groups who peacefully demonstrate against a political entity, government institution, policy, group, tradition, business, or other private institution.”^19^ Protests that are coordinated in some way but occur in different locations or on different days are counted as unique events. Protest events can take a range of forms including marches, sit-ins and strikes involving demonstrations. Examples of protest events can be found in the online repository.^20^ Entries into the Armed Conflict Location and Event Data Project data set are identified by an expert research team that continuously surveys local, national and international media sources (including newspapers and radio); reports of governmental and nongovernmental organizations; and vetted social media sources. Each event included in the data set has details on the categories of people involved, which enabled us to focus on events with health workers. Ethics approval was not required for this observational study of aggregated data.

To study the effect of the pandemic on protest activity, we identified health worker protest events between 2018 and 2022. We restricted our analysis to countries and territories that the Armed Conflict Location and Event Data Project surveyed for the entirety of this time period, which resulted in a data set of 159 countries and territories (Box 1). We put no further restrictions on the jurisdictions included in our modelling so that our analysis could be as globally representative as possible. Taken as a whole, the countries in our sample represent about 85% (6.65 billion/7.84 billion) of the world’s population. We aggregated data to weekly time periods, including a prepandemic period of 114 weeks before and a postpandemic period of 143 weeks after the World Health Organization (WHO) classified COVID-19 as a pandemic on 11 March 2020.

Box 1 **Countries and territories included in the analysis of health worker protests, 2018–2022**

Afghanistan; Albania; Algeria; Angola; Anguilla; Antigua and Barbuda; Argentina; Armenia; Aruba; Azerbaijan; Bahamas; Bahrain; Bangladesh; Barbados; Belarus; Belize; Benin; Bolivia (Plurinational State of); Bosnia and Herzegovina; Botswana; Brazil; British Indian Ocean Territory; British Virgin Islands; Bulgaria; Burkina Faso; Burundi; Cambodia; Cameroon; Caribbean Netherlands; Cayman Islands; Central African Republic; Chad; Chile; China; China, Taiwan; Colombia; Congo; Costa Rica; Côte d'Ivoire; Croatia; Cuba; Curaçao; Cyprus; Democratic People's Republic of Korea; Democratic Republic of Congo; Djibouti; Dominica; Dominican Republic; Ecuador; Egypt; El Salvador; Equatorial Guinea; Eritrea; Eswatini; Ethiopia; Falkland Islands; French Guiana; Gabon; Gambia; Georgia; Ghana; Greece; Grenada; Guadeloupe; Guatemala; Guinea; Guinea-Bissau; Guyana; Haiti; Honduras; India; Indonesia; Iran (Islamic Republic of); Iraq; Israel; Jamaica; Japan; Jordan; Kazakhstan; Kenya; Kosovo; Kuwait; Kyrgyzstan; Lao People's Democratic Republic; Lebanon; Lesotho; Liberia; Libya; Madagascar; Malawi; Malaysia; Mali; Martinique; Mauritania; Mexico; Mongolia; Montenegro; Montserrat; Morocco; Mozambique; Myanmar; Namibia; Nepal; Nicaragua; Niger; Nigeria; North Macedonia; occupied Palestinian territory, including east Jerusalem; Oman; Pakistan; Panama; Paraguay; Peru; Philippines; Puerto Rico; Qatar; Republic of Korea; Republic of Moldova; Romania; Russian Federation; Rwanda; Saint Barthelemy; Saint Kitts and Nevis; Saint Lucia; Saint Martin; Saint Vincent and the Grenadines; Saudi Arabia; Senegal; Serbia; Sierra Leone; Sint Maarten; Somalia; South Africa; South Georgia and the South Sandwich Islands; South Sudan; Sri Lanka; Sudan; Suriname; Syrian Arab Republic; Tajikistan; Thailand; Togo; Trinidad and Tobago; Tunisia; Türkiye; Turkmenistan; Turks and Caicos; Uganda; Ukraine; United Arab Emirates; United Republic of Tanzania; Uruguay; US Virgin Islands; Uzbekistan; Venezuela (Bolivarian Republic of); Viet Nam; Yemen; Zambia; and Zimbabwe.

Our analysis focused on two main outcome measures. First, to investigate the overall number of protest events, we summed the total weekly number of health worker protests across all countries in our data set. This outcome measure reflects the weekly volume of health worker protests in all countries included in our data set. Second, to determine the number of countries that experienced health worker protests, we calculated the number of countries with one or more health worker protests in a given week. This outcome measure reflects the dispersion of health worker protests in countries included in our data set. Both outcome measures consider all protest events as defined by the Armed Conflict Location and Event Data Project, including but not limited to marches, sit-ins and strikes with demonstrations.

### Statistical analysis

We used interrupted time series analysis, a robust quasi-experimental research design. This analysis enables researchers to control for pre-existing trends in time series data by making multiple assessments of an outcome before and after an intervention or external shock.^21^ By controlling for pre-existing trends, interrupted time series analysis facilitates causal inference about the effect of an external shock on outcomes of interest in both the short (immediate level changes) and long term (trend changes). We fitted segmented linear regression using generalized least squares models that included adjustments for autocorrelation based on standard tests for model fit.^21^ We also assessed our models for nonlinearity and included a quadratic trend term in both models as it significantly improved our model fit. To assess whether model specification was driving our results, we also performed a sensitivity analysis using autoregressive integrated moving average regression. We used R version 4.2.1 (R Foundation, Vienna, Austria) for all analyses.

## Results

We identified a total of 18 322 health worker protests in 133 countries during our study period. These protests included different types of health workers – including, but not limited to, nurses, physicians, community health workers and midwives – working in both the public and private sectors. On average, 71.3 protests took place per week, with one or more protests in an average of 25.6 countries in any given week. Health worker protests were concentrated in certain countries. As shown in Table 1, a subset of countries drove most of the protest activity in both the prepandemic period and postpandemic declaration period of our study. Overall, the top 10 countries accounted for 55% (10 009/18 322) of all protest activity. We were unable to conduct interrupted time series modelling at the country or regional level as data for these analyses exhibited substantial random variability and there were small weekly sample sizes. However, visual examination of regional data showed that trends in protest activity were broadly similar across regions, with a more pronounced increase in protest activity in the WHO Region of the Americas and the African and the Eastern Mediterranean Regions at the outset of the pandemic (online repository).^20^

**Table 1:** Top 10 countries for health worker protest activity, 2018–2022

Country	Total no. of protests		Average no. of weekly protests	
			Before COVID-19 declared a pandemic	After COVID-19 declared a pandemic
India	2719		10.5	10.6
Pakistan	1417		5.6	5.5
Mexico	1062		2.9	5.1
Türkiye	948		2.4	4.7
Argentina	869		1.3	5.0
Bolivarian Republic of Venezuela	867		3.5	3.2
Brazil	781		2.3	3.6
Republic of Korea	573		1.7	2.6
Islamic Republic of Iran	400		1.1	1.9
Morocco	373		0.9	1.9

COVID-19: coronavirus disease 2019.

### Total weekly protests

**Fig. 1:**
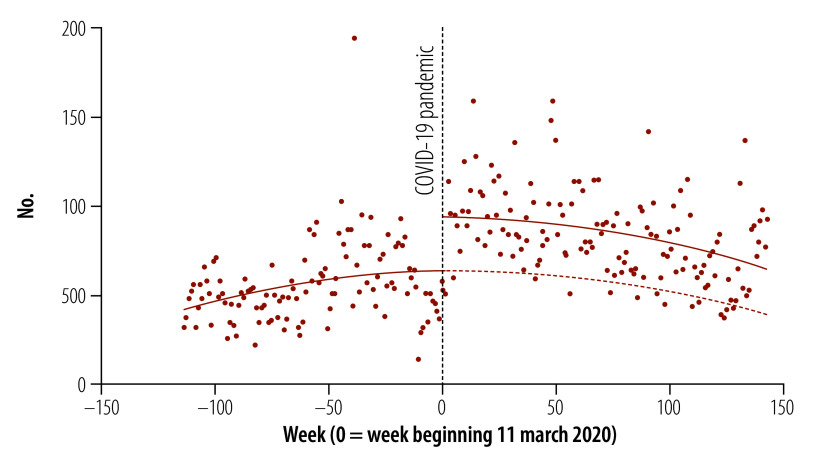
Total health worker protest activity globally, 2018–2022

The results of our interrupted time series analysis of total weekly protest activity are shown in Fig. 1. We found an immediate level increase of 30.1 additional health worker protests per week globally after WHO classified COVID-19 as a pandemic (95% confidence interval, CI: 11.7–48.6). This level of protest activity was 47% (30.1/63.5) higher than would have been expected based on the prepandemic trajectory. We also found no significant change in the trend, suggesting that this increase in health worker protest activity was sustained over our study period. Our sensitivity analysis using autoregressive integrated moving average modelling found similar results (online repository).^20^

COVID-19: coronavirus disease 2019.

Note: The outlying point at week 76 in the prepandemic period is driven by a wave of protests in India between 12 and 18 June 2019. The dashed line represents the counterfactual scenario: that is, an extrapolation of the trend in protest activity before COVID-19 was declared a pandemic into the period after COVID-19 was declared a pandemic, and reflects what would be expected in the absence of an external shock.

### Protest activity across countries

**Fig. 2:**
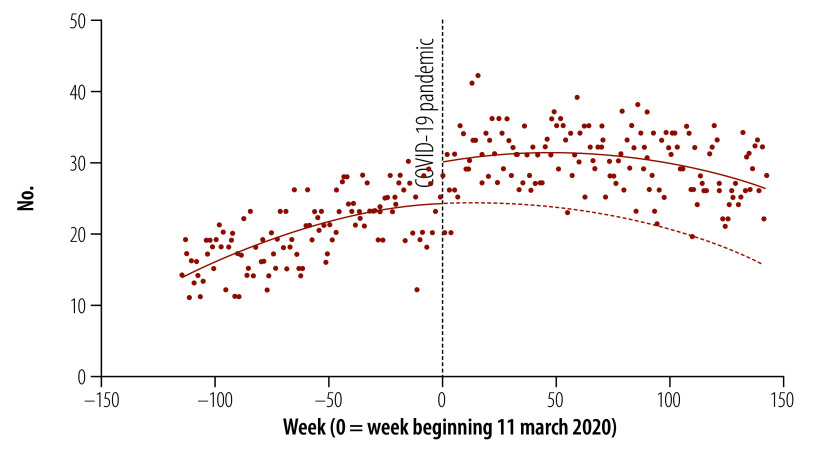
Number of countries and territories with at least one health worker protest in a given week globally, 2018–2022

Similar to overall protest activity, the COVID-19 pandemic was associated with an increase in the number of countries that experienced health worker protests. As shown in Fig. 2, after WHO’s declaration of a pandemic, we observed a significant and sustained increase in the number of countries with at least one health worker protest in a given week (level increase of 5.7; 95% CI: 3.5–7.8). This result represented a 24% (5.7/24.1) increase in the number of countries experiencing protests in a given week compared with what would be expected if prepandemic trends persisted. As with the overall number of protest events, we found no significant change in the trend of this outcome. The sensitivity analysis showed similar results using autoregressive integrated moving average modelling (online repository).^20^

COVID-19: coronavirus disease 2019.

Note: The dashed line represents the counterfactual scenario, that is an extrapolation of the trend in protest activity before COVID-19 was declared a pandemic into the period after COVID-19 was declared a pandemic, and reflects what would be expected in the absence of an external shock.

## Discussion

The COVID-19 pandemic has led to major shifts in and pressures on health systems around the world. Our analysis, which is based on data covering 85% of the world’s population, indicates that the pandemic accelerated an already increasing frequency of health worker protests globally. We found this result with two different indicators of health worker protest activity: weekly protest activity by health workers rose after 11 March 2020 compared with the expected trajectory, and the number of countries with at least one protest in a given week increased. Collectively, these results indicate that the COVID-19 pandemic was associated with an increased frequency and spread of protests by health workers. These findings provide empirical support for previous claims that health worker protests increased because of the COVID-19 pandemic. As a manifestation of structural weaknesses in health systems, health worker protests are important to track and investigate. Taken together, health worker protests signal systemic challenges, such as underinvestment, poor working conditions and lack of emergency preparedness, that can negatively affect patient health outcomes and access to care. Although these challenges predate the pandemic, they gained new urgency after its onset. In the time since our study period, many protest actions by health workers have taken place around the world (for example, strikes by nurses, ambulance drivers and junior doctors in the United Kingdom of Great Britain and Northern Ireland in 2023 and 2024), indicating that protest action is still a key tool for expressing grievances about health system challenges.^22^

The use of protests by health workers has gained visibility in recent years, likely driven in part by the struggles experienced by health workers during the pandemic. Before the pandemic, interest in the use of protests, and particularly strikes, by health workers was growing in global health-policy communities, particularly in the context of work stoppages in low- and middle-income countries.^23^ Prepandemic estimates of global health worker protest activity found an increasing frequency of health worker protests in 23 low-income countries.^15^ Furthermore, prior analyses of health worker protests during the COVID-19 pandemic (which have had methodological weaknesses) associated the onset of the pandemic with a rise in health worker protests.^16^^,^^17^ The direction of the findings from these earlier studies corresponds with our results. The important strengths of our paper that increase our confidence in our findings include the use of interrupted time series analysis, which enabled us to account for pre-existing trends in the data, and the use of the Armed Conflict Location and Event Data Project data set, which provided comprehensive and systematically collected data on protest activity in a wider range of countries than has been previously examined.

The implications of these protest events for governments, health systems and, most importantly, patients and communities need further investigation. A meta-analysis on the effect of strikes on patient outcomes did not provide significant evidence that strikes affect in-hospital or population mortality.^7^ However, this evidence was variable and came mostly from high-income countries, and notable case studies have been published that show higher rates of patient mortality during strike periods.^4^ Patient outcomes during periods of labour action are likely moderated by the availability of so-called safety nets to ease disruptions to service delivery, although these safety nets are more limited in some low- and middle-income countries.^8^ The volume of protest activity observed globally in our study suggests that decision-makers must pay attention to the structural failings within health systems that are driving these protests, and the harms experienced by patients and workers when these failings are not addressed.

Exploring the drivers of protest events is important to interpret these data and develop policy solutions. The quantifiable increase in health worker protests we identified is complemented by existing but nascent research exploring these drivers.^11^^,^^13^^,^^24^ The 2022 study mentioned previously reported that 66% (4 358/6 589) of health worker protests in the first year of the pandemic were about working conditions and remuneration.^17^ This study also found that only half of the protests in the first year of the pandemic were directly related to COVID-19, highlighting that issues that predated the pandemic were still relevant to workers during this time. A study of health and retail workers in 90 countries identified that pay concerns, followed closely by health and safety concerns, were the main drivers of protests in the health sector during the first year of the COVID-19 pandemic.^16^ This study highlights that the scale of non-pandemic-related protests indicates that structural challenges in health systems drove protest action more than the immediate threats of COVID-19. The study also found substantially more protest activity among health workers than retail workers, potentially due to higher levels of union membership and specialization. In summary, two factors might help explain the increase in protests we observed. First, health workers experienced extraordinary pressure during the pandemic that was exacerbated by existing structural health system failings predating the pandemic. Second, the attention that health workers were able to secure from governments and the public during the pandemic period – particularly given the public pronouncements of support – made this period an important moment for health workers to voice their demands.

Our design and analysis have some limitations. Importantly, our country sample included 159 countries and territories which were primarily low- and middle-income. While this data set is the most comprehensive available to study this topic, our results may not be generalizable across all countries and territories. Furthermore, due to the nature of our data set, we were limited to analysing only two outcomes: total weekly protests and the number of countries with at least one protest in any given week. Future studies that use different data sources or engage in primary data collection can help answer outstanding questions about the protest events, for example, the forms that protest actions take and the demands made. A final potential limitation is our a priori use of a quadratic term in our interrupted time series models, which resulted in the predicted counterfactual line to curve downwards after the onset of the pandemic. However, given that we present results based on an immediate level change, we do not believe that modifying our modelling approach would change our interpretation.

Our findings have implications for research, policy and practice. The evidence on global trends in health worker protests is still limited and further analysis is needed to understand the drivers of protests, the types of health worker engaged in protest action and the shifts in the geographic distribution of protests. Research on specific protest events or clusters of protest events, including the role of protest in governance processes, is needed, particularly in low- and middle-income countries where protest activity appears to have accelerated.^15^ Further research is also needed on the effect that factors such as sex, race, ethnicity, class and caste have on the use of protest to vocalize grievances. From a policy standpoint, the sharp increase in the level of protest after the onset of the pandemic is at odds with widespread public pronouncements of support for health workers, particularly in the early phases of the pandemic. Therefore, governments and employers (public and private) should commit to greater investments in the health workforce to ensure universal health coverage goals are met and health systems are resilient to shocks, such as pandemics. Furthermore, governments and employers should prioritize support for and engagement of health workers when devising future pandemic preparedness plans. Finally, the prevalence of health worker protests we identified in our study suggests that more inclusive platforms and mechanisms in health workforce governance – especially mechanisms that ensure voice and representation for different groups in the health workforce – are needed to facilitate timely and responsive decision-making about key health system challenges.

## References

[1] Boniol M, Kunjumen T, Nair TS, Siyam A, Campbell J, Diallo K. The global health workforce stock and distribution in 2020 and 2030: a threat to equity and “universal” health coverage? BMJ Glob Health. 2022 Jun;7(6):e009316. 10.1136/bmjgh-2022-009316 35760437 PMC9237893

[2] The Lancet Global Health. Health-care workers must be trained and retained. Lancet Glob Health. 2023 May;11(5):e629. 10.1016/S2214-109X(23)00172-9 37061296

[3] Essex R, Weldon SM. Health care worker strikes and the Covid pandemic. N Engl J Med. 2021 Jun 17;384(24):e93. 10.1056/NEJMp2103327 33826818

[4] Gruber J, Kleiner SA. Do strikes kill? Evidence from New York state. Am Econ J Econ Policy. 2012;4(1):127–57. 10.1257/pol.4.1.127

[5] Ong’ayo G, Ooko M, Wang’ondu R, Bottomley C, Nyaguara A, Tsofa BK, et al. Effect of strikes by health workers on mortality between 2010 and 2016 in Kilifi, Kenya: a population-based cohort analysis. Lancet Glob Health. 2019 Jul;7(7):e961–7. 10.1016/S2214-109X(19)30188-3 31129126 PMC6560003

[6] Sim J, Choi Y, Jeong J. Changes in emergency department performance during strike of junior physicians in Korea. Emerg Med Int. 2021 Jul 8;2021:1786728. 10.1155/2021/1786728 34306757 PMC8285189

[7] Essex R, Weldon SM, Thompson T, Kalocsányiová E, McCrone P, Deb S. The impact of health care strikes on patient mortality: a systematic review and meta-analysis of observational studies. Health Serv Res. 2022 Dec;57(6):1218–34. 10.1111/1475-6773.14022 35791855 PMC9643090

[8] Essex R, Brophy SA, Sriram V. Strikes, patient outcomes, and the cost of failing to act. BMJ. 2023 Mar 10;380:e072719. 10.1136/bmj-2022-072719 36898728

[9] Ameso EA, Prince RJ. Striking health workers: precarity and healthcare in neoliberal Kenya. Anthropol Today. 2022;38(4):11–4. 10.1111/1467-8322.12742

[10] Binkowska-Bury M, Marc M, Nagorska M, Januszewicz P, Ryzko J. The opinions of Polish nurses and patients on nursing protests. Coll Antropol. 2013 Sep;37(3):691–9. 24308205

[11] Koon AD. When doctors strike: making sense of professional organizing in Kenya. J Health Polit Policy Law. 2021 Aug 1;46(4):653–76. 10.1215/03616878-8970867 33493308

[12] Khan A. Lady health workers and social change in Pakistan. Econ Polit Wkly. 2011;46(30):28–31.

[13] Polak P, Wagner A, Świątkiewicz-Mośny M. Our good is the public good – reframing the communication of professional groups. Anatomy of the resident doctors’ protests in Poland. Soc Mov Stud. 2022;21(3):274–96. 10.1080/14742837.2020.1865906

[14] Hardy J, Calveley M, Kubisa J, Shelley S. Labour strategies, cross-border solidarity and the mobility of health workers: evidence from five New Member States. Eur J Ind Relat. 2015;21(4):315–33. 10.1177/0959680114553159

[15] Russo G, Xu L, McIsaac M, Matsika-Claquin MD, Dhillon I, McPake B, et al. Health workers’ strikes in low-income countries: the available evidence. Bull World Health Organ. 2019;97(7):460-467H. 10.2471/BLT.18.225755 31258215 PMC6593336

[16] Trapmann V, Umney C, Neumann D, Stuart M, Joyce S, Bessa I. Labour protests during the pandemic: the case of hospital and retail workers in 90 countries. Geneva: International Labour Organization; 2022. 10.54394/WEVN1114

[17] Brophy S, Sriram V, Zong H, Andres C, Mawyin M. GL N. Heroes on strike: trends in global health worker protests during COVID-19. Washington, DC: Accountability Research Center; 2022.

[18] Raleigh C, Linke A, Hegre H, Karlsen J. Introducing ACLED: an armed conflict location and event dataset. J Peace Res. 2010;47(5):651–60. 10.1177/0022343310378914

[19] Armed conflict location & event data project (ACLED) codebook [internet]. ACLED; 2023. Available from: https://acleddata.com/knowledge-base/codebook/ [cited 2024 May 12].

[20] Sharma K, Brophy S, Law M, Sriram V. Global health worker protests and the COVID-19 pandemic: an interrupted time-series analysis [online repository]. Charlottesville: Center for Open Science; 2024. 10.17605/OSF.IO/JDGFN 10.17605/OSF.IO/JDGFN PMC11362691 39219771

[21] Penfold RB, Zhang F. Use of interrupted time series analysis in evaluating health care quality improvements. Acad Pediatr. 2013 Nov-Dec;13(6) Suppl:S38–44. 10.1016/j.acap.2013.08.002 24268083

[22] Torjesen I. Doctors’ strikes one year on: what will it take to end the disputes? BMJ. 2024 Mar 8;384:q591. 10.1136/bmj.q591 38458642

[23] Salama P, McIsaac M, Campbell J. Health workers’ strikes: a plea for multisectoral action. Bull World Health Organ. 2019 Jul 1;97(7):443–443A. 10.2471/BLT.19.238279 31258210 PMC6593339

[24] Mishra A, Elias MA, Sriram V. A Draconian law: examining the navigation of coalition politics and policy reform by health provider associations in Karnataka, India. J Health Polit Policy Law. 2021 Aug 1;46(4):703–30. 10.1215/03616878-8970895 33493290

