# Corrigendum

**DOI:** 10.2471/BLT.24.101124

**Published:** 2024-11-01

In: Sharma K, Sorcha B, Law M, Sriram V, Health worker protests and the COVID-19 pandemic: an interrupted time-series analysis. Bull World Health Organ. 2024 Sep 1; 102 (9): 650–6., 

on page 652, Figure 1 should appear as follows:

**Fig. 1:**
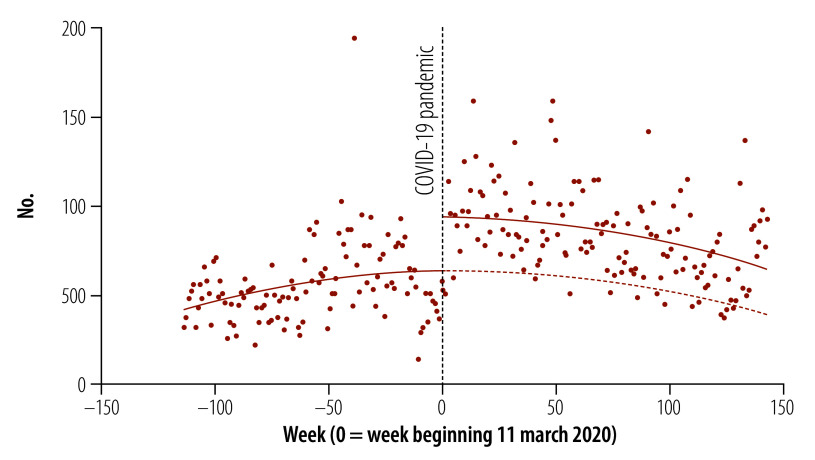
Total health worker protest activity globally, 2018–2022

COVID-19: coronavirus disease 2019.

Note: The outlying point at week 76 in the prepandemic period is driven by a wave of protests in India between 12 and 18 June 2019. The dashed line represents the counterfactual scenario: that is, an extrapolation of the trend in protest activity before COVID-19 was declared a pandemic into the period after COVID-19 was declared a pandemic, and reflects what would be expected in the absence of an external shock.

